# Induction of Defense Responses and Partial Control of Powdery Mildew and Gray Mold in *Vitis vinifera* cv. Chardonnay by *Pseudomonas protegens*-Based Formulations

**DOI:** 10.3390/plants15091371

**Published:** 2026-04-30

**Authors:** Braulio Ruiz, Mauricio Sanz, Yerko Lovera, Juan San Martín, Felipe Gaínza-Cortés, Ernesto Moya-Elizondo

**Affiliations:** 1 Laboratorio de Fitopatología, Departamento de Producción Vegetal, Facultad de Agronomía, Universidad de Concepción, Av. Vicente Méndez 595, Chillán 3812120, Chile; braruiz@udec.cl (B.R.); msanz2016@udec.cl (M.S.); ylovera@udec.cl (Y.L.); juasanmartinm@udec.cl (J.S.M.); 2 Center for Research and Innovation, Viña Concha y Toro S.A., Fundo Pocoa s/n, Km10 Ruta K-650, Pencahue 3550000, Chile; felipe.gainza@conchaytoro.cl

**Keywords:** *Botrytis cinerea*, *Erysiphe necator*, biological control, defense-related genes, plant immunity, grapevine integrated disease management

## Abstract

Grapevine (*Vitis vinifera* L.) is an economically important fruit crop cultivated worldwide. However, its production and fruit quality are severely constrained by powdery mildew (*Erysiphe necator*) and Botrytis bunch rot (*Botrytis cinerea*) diseases. Increasing concerns regarding chemical fungicide resistance and environmental sustainability highlight the urgent need to develop alternative and more sustainable disease management strategies. This study assessed the field efficacy of *Pseudomonas protegens*-based formulations (TANIRI^®^ WP at 1 g·L^−1^ and MaxGrowth at 1 mL·L^−1^) within an integrated disease management program in cv. Chardonnay. Defense-related gene expression analysis revealed that biological treatments predominantly up-regulated *pr1*, *pr2*, and *pr10* in both leaves and berries. In contrast, the chemical inducer acibenzolar-S-methyl (ASM) triggered earlier but less consistent induction of *pr1* and *pr2*, alongside transient activation of *pal* and *lox9*. Repeated field applications of *P. protegens* formulations moderately reduced the severity of Botrytis bunch rot (20.89%) and powdery mildew (6.14%), though control levels remained below conventional sulfur/*Bacillus subtilis*-based treatments (30.04% and 13.56%, respectively). Overall, these findings suggest that biological inducers could complement conventional management practices for grapevine health. In particular, *P. protegens* may act mainly by systemically inducing host defense responses and partially suppressing pathogen development under field conditions.

## 1. Introduction

Grapevine (*Vitis vinifera* L.) ranks among the foremost fruit crops worldwide, playing a central role in agriculture and global wine industry [1]. In Chile, vineyards are a major component of the national fruit sector; according to the National Viticultural Cadastre, 124,436 hectares are currently planted for wine production. In 2024, Chilean wine exports reached approximately 777 million liters, underscoring the sector’s substantial contribution to agribusiness and export revenues [2]. Nevertheless, grapevines are frequently affected by the fungus *Erysiphe necator* (Schwein.) Burrill, the causal agent of powdery mildew, and *Botrytis cinerea* Pers.: Fr., responsible for Botrytis bunch rot. These diseases remain among the most persistent and economically significant threats in viticulture worldwide [3,4,5,6].

Powdery mildew is one of the most destructive pre-harvest diseases globally, reducing bunch weight, delaying fruit ripening, impairing photosynthesis, and compromising grape and wine quality by altering soluble solids, pigmentation, and aromatic profiles [7]. Botrytis bunch rot further threatens yield and post-harvest quality, particularly under conducive environmental conditions such as humid climates [8]. Conventional disease management relies primarily on repeated fungicide applications throughout the growing season, from flowering to harvest. However, this practice raises environmental concerns [6,9] and contributes to the emergence of fungicide resistance in both grapevine pathogens [10,11,12]. These phytosanitary challenges underscore the need for innovative and environmentally sustainable disease management strategies, in which interactions between plants and beneficial microorganisms represent a promising approach to reduce disease losses and ecological impacts [13,14,15,16,17].

Among biological control agents, *Pseudomonas protegens* has been recognized for combining direct antagonistic activity with the ability to stimulate plant defense responses [18,19,20]. Its relevance to the management of powdery mildew and Botrytis bunch rot in grapevine is supported by three complementary mechanisms. First, *P. protegens* produces antifungal metabolites such as 2,4-diacetylphloroglucinol, pyoluteorin, and hydrogen cyanide, which are active against filamentous fungi and may directly inhibit *E. necator* and *B. cinerea* during early stages of infection [21,22]. Second, competition for nutrients and iron through siderophore production can limit pathogen establishment on plant surfaces [23,24]. Third, *P. protegens* can activate host defense responses, thereby enhancing the plant’s capacity to restrict pathogen colonization [22,25]. These mechanisms are particularly relevant for powdery mildew and Botrytis bunch rot in grapevine, as both pathogens depend on rapid surface colonization and successful penetration of host tissues, processes that can be constrained by pre-activated chemical and structural defenses [18,23].

*P. protegens* has been widely reported as a beneficial bacterium capable of activating plant defense responses. In *Arabidopsis thaliana*, several studies have shown that *P. protegens* triggers transcriptional changes associated with salicylic acid (SA) and jasmonic acid (JA) dependent defense pathways, resulting in enhanced resistance to subsequent pathogen attack [26,27]. This transcriptional activation reflects an induced state of resistance that increases the plant’s defensive capacity upon challenge. Despite this growing body of evidence in model plants, the capacity of *P. protegens* to induce resistance in fruit crops remains poorly characterized, with only a limited number of studies reporting beneficial effects in apple and peach [28,29,30].

Induced resistance (IR) refers to a physiological state in which plant defense mechanisms are activated by biological or chemical stimuli, leading to enhanced protection against a broad range of pathogens [20,31,32]. This activated state is not restricted to the site of induction but can extend systemically throughout the plant, a phenomenon classically described as systemic acquired resistance (SAR) [33]. At the molecular level, IR is associated with extensive transcriptional reprogramming and the accumulation of defense-related components, including pathogenesis-related proteins, enzymes of secondary metabolism, and compounds involved in cell wall reinforcement [11,31,34,35]. These changes enable plants to respond more effectively to pathogen invasion and constitute a key mechanism underlying durable, broad-spectrum disease control [36].

In grapevine, IR-based strategies have recently been associated with the partial control of powdery mildew through the application of beneficial bacteria, such as *Pseudomonas fluorescens* CHA0 [37] and *Bacillus* spp. strains Buz14 and S38 [38]. However, to date, no studies have reported IR triggered by *P. protegens* in grapevine. In Chile, native strains of *P. protegens* have been extensively evaluated in other cropping systems, including wheat [39,40,41,42], industrial chicory [43], hazelnut [44], and kiwifruit, where they have shown both antagonistic activity and the ability to induce defense-related gene expression against *Pseudomonas syringae* pv. *actinidiae* [45,46]. Recently, different formulations based on these native strains have been developed for plant disease management; nevertheless, their capacity to induce resistance and contribute to the control of powdery mildew or Botrytis bunch rot in grapevine remains unexplored.

This work aimed to evaluate the induction of grapevine defense genes by *P. protegens*-based formulations, in comparison with acibenzolar-S-methyl (ASM). The study focused on the expression of key defense-related genes in grapevine, including SA-responsive genes (*pr1*, *pr2*, and *pr10*), genes associated with JA biosynthesis (*lox9*), and a central gene of the phenylpropanoid pathway (*pal*). These genes are commonly used as molecular markers of defense activation in grapevine–pathogen interactions. Additionally, this work determined whether treatment with *P. protegens* strains and *P. protegens*-based formulations reduced disease severity and incidence of powdery mildew and Botrytis bunch rot across different experimental systems, including leaf discs, grape berries, and field trials.

## 2. Results

### 2.1. Results of Relative Expression Assays to Determine Defense Gene Induction

**Figure 1:**
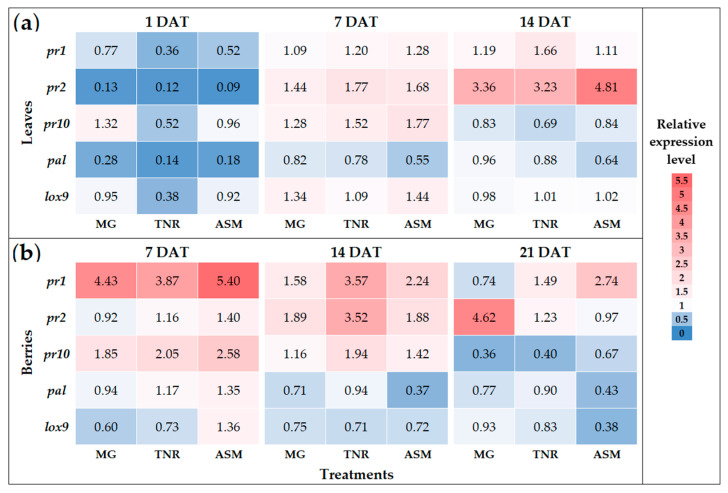
Heat map representation of the relative expression levels of five defense-related genes (*pr1*, *pr2*, *pr10*, *pal*, and *lox9*) in grapevine tissues following treatment with biological and chemical inducers. Expression profiles were analyzed in grapevine leaves at 1, 7, and 14 days after treatment (DAT) (**a**) and in grapevine berries at 7, 14, and 21 DAT (**b**). Transcript levels were normalized using the reference genes *act* and *ubc* and are expressed relative to the untreated control (UTC). Heat map values represent median fold-change values derived from independent biological replicates for each treatment and time point. Treatments included MG (MaxGrowth), TNR (TANIRI^®^ WP), and ASM (BION^®^ 50 WG). Color intensity indicates relative expression levels, with a gradient from dark blue (lower expression) to dark red (higher expression).

The temporal expression patterns of defense-related genes in grapevine leaves and berries following treatment with *P. protegens*-based formulations TANIRI^®^ WP (TNR) and MaxGrowth (MG) and the chemical inducer ASM are summarized in Figure 1 and Appendix A Table A1 and Table A2.

In leaves (Figure 1a), genes associated with pathogenesis-related responses exhibited the most pronounced transcriptional modulation. The expression of *pr1* showed a slight increase at 7 days after treatment (DAT) under all treatments and remained marginally above basal levels at 14 DAT, with fold-change values generally ranging between approximately 1.1 and 1.7 relative to the untreated control (UTC), indicating a modest and sustained upward trend over time. In contrast, *pr2* displayed a marked time-dependent response. Although expression levels were strongly repressed at 1 DAT (fold change < 0.2 across treatments), a clear induction was observed at later stages, particularly at 14 DAT, when ASM reached the highest expression (fold change ≈ 4.8), followed by MG and TNR (≈3.3). This pattern suggests a delayed activation of *pr2* in leaves, with differences in magnitude among inducers. The *pr10* transcript exhibited a transient induction profile in leaves, with moderate increases at 7 DAT (fold change between ~1.5 and 1.8) and a subsequent decline to basal or sub-basal levels at 14 DAT. The expression of *pal* was characterized by an early repression at 1 DAT (fold change < 0.3 in all treatments) and a partial recovery at later time points, without exceeding basal levels. For *lox9*, only moderate and transient increases were detected, with the highest value observed at 7 DAT under ASM treatment (fold change ≈ 1.4), whereas MG and TNR remained close to basal expression throughout the experiment.

In grapevine berries (Figure 1b), transcriptional responses were more pronounced for *pr* genes and differed in timing compared to leaves. The *pr1* gene showed strong early induction at 7 DAT, particularly under ASM (fold change ≈ 5.4) and MG (≈4.4), followed by a progressive decline at 14 and 21 DAT, indicating a rapid but transient response in fruit tissues. In contrast, *pr2* exhibited a delayed induction pattern under biological treatments, with MG reaching its highest expression at 21 DAT (fold change ≈ 4.6), and TNR peaking at 14 DAT (fold change ≈ 3.5). ASM promoted earlier *pr2* induction (fold change ≈ 1.4–1.9 at 7–14 DAT), which was not sustained in the later stage. The expression of *pr10* in berries was transient across all treatments, with maximum induction at 7 DAT (fold change ≈ 2.6 under ASM and ≈2.0 under MG and TNR), followed by a marked decrease at 14 and 21 DAT. In contrast, *pal* and *lox9* did not show consistent induction under biological treatments, remaining close to or below basal levels. ASM induced only modest and short-lived increases in these genes, such as a transient *lox9* elevation at 7 DAT (fold change ≈ 1.36), which declined thereafter.

Overall, Figure 1b illustrates distinct, tissue-specific transcriptional trends in response to biological and chemical inducers. *P. protegens*-based formulations preferentially promoted the delayed and sustained induction of *pr* genes in berries, particularly *pr2*, whereas ASM generally triggered earlier responses of greater initial magnitude but with limited persistence over time. These patterns indicate differential temporal modulation of assessed grapevine defense-related genes depending on tissue type and inducer.

### 2.2. Results of Leaf Disc Assays Evaluating Pseudomonas protegens-Induced Control of Botrytis cinerea and Erysiphe necator in Grapevine

**Figure 2:**
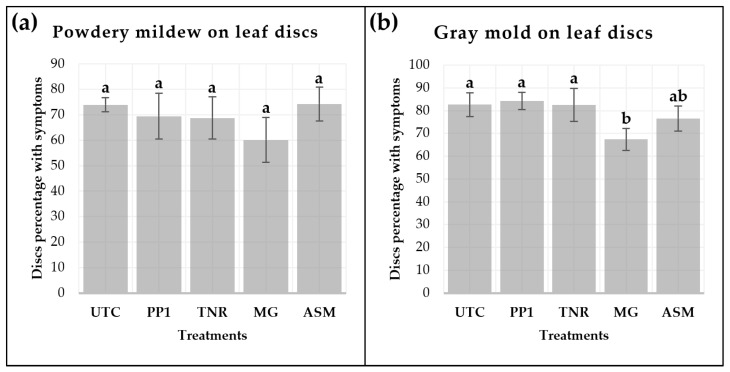
Severity of *Erysiphe necator* on leaf discs (**a**) and severity of *Botrytis cinerea* infection on leaf discs (**b**), measured as the percentage of leaf surface area affected. Treatments included a control treated with distilled water (UTC), *Pseudomonas protegens* strains Ca2 and ChC7 (PP1), a dust formulation (TANIRI^®^ WP [TNR]), a liquid suspension formulation (MaxGrowth [MG]), and acibenzolar-S-methyl (BION^®^ 50 WG [ASM]). Leaf discs were assessed 7 days after inoculation and maintained at 25 °C [47]. Error bars represent the standard error (SE) of the mean between biological replicates (*n* = 12). Statistical differences in panel (**b**) are indicated by different letters above the bars, based on Tukey’s HSD test (*p* ≤ 0.05).

Chardonnay leaf discs, previously treated in the field with *P. protegens*-based formulations and ASM and subsequently inoculated in the laboratory with *B. cinerea* and *E. necator*, exhibited variable levels of disease severity compared with the SDW-treated control (Figure 2). For powdery mildew, no statistically significant differences among treatments were detected. Disease severity caused by *E. necator* averaged 74.0% in the untreated control (UTC; Figure 2a). Treatments with *P. protegens* strains (PP1) and its formulations (TNR, MG) showed numerically lower mean severity levels (60.1–69.0%), with MG exhibiting the lowest percentage of observed symptoms, although these reductions were not statistically significant (*p* > 0.05).

In contrast, for *B. cinerea*, the MG treatment significantly reduced leaf disc severity compared to UTC (*p* ≤ 0.05), while ASM displayed a non-significant but numerically lower mean severity (Figure 2b). Specifically, MG achieved a 15.31% reduction relative to UTC, whereas ASM showed a 6.15% reduction.

### 2.3. In Vivo Assessment of the Efficacy of Microbial and Chemical Resistance Inducers on Grapevine Berries Inoculated with Botrytis cinerea

**Figure 3:**
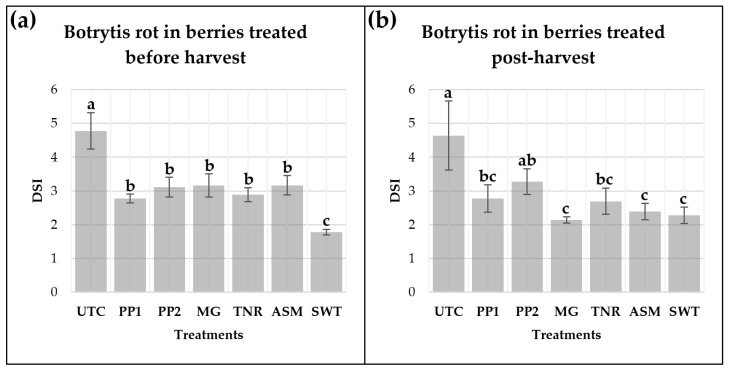
The disease severity index (DSI) of *Botrytis cinerea* (Bc) infection in grape berries treated with *Pseudomonas protegens* strains (PP1, PP2) and their formulations (MG, TNR), acibenzolar-S-methyl (ASM), and a cyprodinil + fludioxonil fungicide mixture (SWT). Treatments were applied in a Chardonnay vineyard seven days prior to harvest, followed by artificial inoculation with Bc under field conditions (**a**), or applied post-harvest and subsequently inoculated with Bc under laboratory conditions (**b**). Disease severity was evaluated after 5 days of incubation in humidity chambers maintained at 21 ± 1 °C. Six healthy berries were selected from each bunch and placed in an individual humid chamber, which contained the assessed seven treatments. Four humid chambers per plot were assessed to obtain the plot mean. Disease severity was scored using the scale described by Herrera-Défaz et al. [48]. Error bars represent the standard error (SE) of the mean. Different letters indicate significant differences according to Tukey’s HSD test (*p* ≤ 0.05).

Two independent bioassays were conducted to evaluate the effect of the treatments on *B. cinerea* infection in Chardonnay grapevine berries. In the first assay, treatments were applied to grape bunches in the vineyard seven days prior to harvest, followed by artificial inoculation with *B. cinerea* under field conditions (Figure 3a). In the second assay, individual berries were treated post-harvest and subsequently inoculated with *B. cinerea* under laboratory conditions (Figure 3b). In both assays, grape bunches were incubated in humidity chambers, and the application of bacteria-based formulations to the berries resulted in a reduction in disease severity.

Disease severity was assessed five days post-harvest, and significant differences among treatments were observed in the first bioassay (Figure 3a). The untreated control (UTC) exhibited the highest severity index and differed significantly from all other treatments (*p* ≤ 0.05). Treatments with a suspension of *P. protegens* strain Ca6 (PP2) and a mixture of Ca2 and ChC7 strains (PP1), as well as MG, TNR, and ASM, showed a moderate reduction in disease severity compared with the control, although no significant differences were detected among them (Figure 3a). In contrast, the cyprodinil + fludioxanil- based fungicide treatment (SWT) showed the lowest severity index among all treatments (*p* ≤ 0.05).

The second bioassay provided a complementary assessment of treatment efficacy under controlled post-harvest conditions (Figure 3b). Consistent with the first assay, the untreated control (UTC) exhibited the highest disease severity index (DSI) confirming the high susceptibility of Chardonnay berries to *B. cinerea* in the absence of protective treatments and differing significantly from most treatments (*p* ≤ 0.05). The application of *P. protegens* strain Ca6 (PP2) resulted in a moderate reduction in disease severity (mean DSI = 3.28); however, this effect was more variable and not statistically different from the control or from the remaining treatments. In contrast, berries treated with the *P. protegens* consortium (PP1), MG, TNR, the chemical inducer ASM, and SWT consistently displayed lower severity indices, ranging between 2.11 and 2.78, indicating a marked reduction in symptom development relative to UTC.

### 2.4. Field Evaluation of Biocontrol Efficacy of Pseudomonas protegens Against Powdery Mildew and Botrytis Bunch Rot

#### 2.4.1. Control of Powdery Mildew on Leaves Under Field Conditions by *Pseudomonas protegens*-Based Formulations

**Figure 4:**
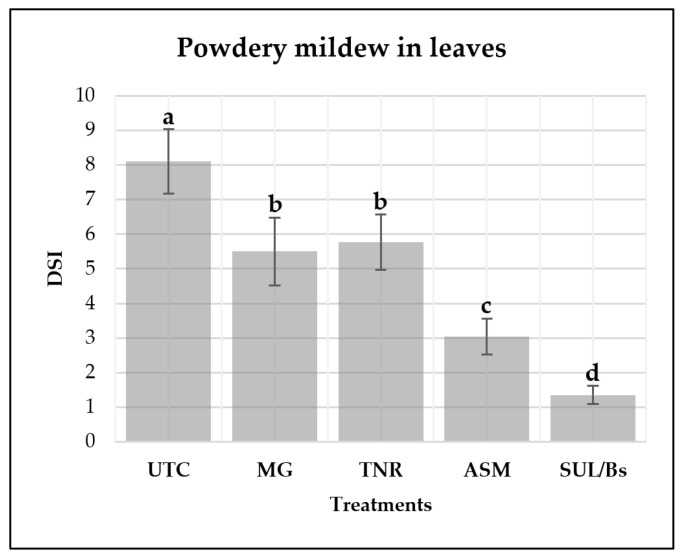
The powdery mildew disease severity index (DSI) on grapevine leaves evaluated at the EL 31 stage (BBCH 75) after four spray treatments. Disease severity was visually assessed using the scale described by Barba et al. [50]: 0 = absent, 1 ≤ 5 small spots, 2 = 5–20 expanding spots, and 3 = widespread powdery mildew with dense sporulation. Treatments included an untreated control (UTC), MaxGrowth (MG), TANIRI^®^ WP (TNR), acibenzolar-S-methyl (ASM), and sulfur/*Bacillus subtilis* QST-713 (SUL/Bs). Data represent mean DSI values (%) per treatment across six replicates. Fifty leaves per grapevine were randomly selected from three central grapevines within each plot (150 leaves per replicate or plot). Different letters indicate significant differences among treatments according to Tukey’s HSD test (*p* ≤ 0.05).

Field evaluations of powdery mildew caused by *E. necator* (Figure 4) revealed significant differences in disease severity among treatments (*p* ≤ 0.05). The vineyard testing area experienced a high level of infection early in the season, providing favorable conditions for evaluating treatment efficacy. The untreated control (UTC) consistently exhibited the highest severity index, confirming the high susceptibility of grapevines to powdery mildew in the absence of control measures [49].

In contrast, all applied treatments reduced disease severity to varying degrees. MG and TNR produced moderate reductions compared to the control, corresponding to decreases of 32.2 and 28.8%, respectively. Notably, ASM and sulfur/*Bacillus subtilis* QST-713 (SUL/Bs) achieved the lowest severity indices (3.04 and 1.35, respectively), reflecting a more pronounced suppression of *E. necator* infection. These reductions correspond to decreases of 62.6% and 83.3%, respectively, relative to the untreated control.

Overall, the comparative treatment performance indicates that while MG and TNR provided partial protection, ASM and SUL/Bs consistently conferred the highest level of control against powdery mildew under field conditions.

#### 2.4.2. Efficacy Assay of *Pseudomonas protegens* to Control Powdery Mildew and Botrytis Bunch Rot in Chardonnay Grapevines Bunches Under Field Conditions

**Figure 5:**
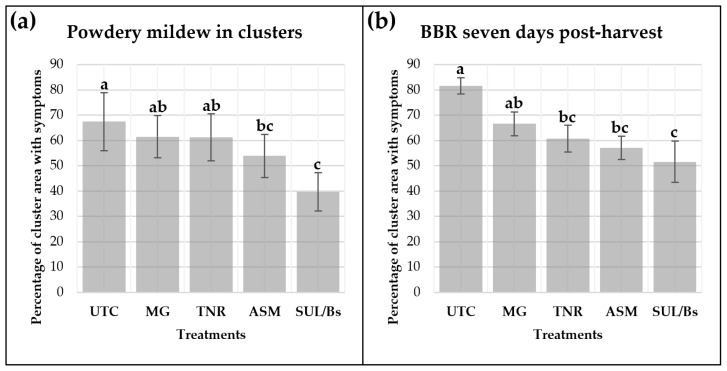
Efficacy of the control of natural infection of powdery mildew (**a**) and Botrytis bunch rot (**b**) on Chardonnay grape bunches using biological and chemical bioinducers. Disease severity on grape bunches, expressed as the percentage of organs with visible disease signs or symptoms was assessed by visual inspection. The graph shows the mean percentage of bunches with symptoms for each treatment, with error bars representing standard errors (SE) among biological replicates. Treatments included an untreated control (UTC), MaxGrowth (MG), TANIRI^®^ WP (TNR), and BION^®^ 50 WG (ASM) and chemical/biological program based in six spraying of Sulfur, followed of four spraying of *Bacillus subtilis* QST-713 (SUL/Bs). Powdery mildew severity was assessed at the EL 31 stage after four initial treatments sprayings (30 bunches per plot), while *Botrytis cinerea* severity was assessed on harvest grape bunches (EL38) following ten sprays of the treatment along the season, and after 7 days of incubation in humidity chambers at 25 °C (18 bunches per plot). Different letters in each panel indicate significant differences among treatments according Tukey’s HSD test (*p* ≤ 0.05).

Powdery mildew control in grape bunches was assessed after four sprayings of the treatments in the season, indicated a reduction in disease severity in some treatments (Figure 5a). Treatments MaxGrowth (MG) and TANIRI^®^ WP (TNR) showed intermediate values and were not significantly different from the UTC. Four sprayings of ASM and sulfur, considered in the SUL/Bs program, reduced significantly the powdery mildew on the bunches at EL 31 stage (53.9% and 39.7% of reduction respect the UTC, respectively).

The results of the control of natural infection by *B. cinerea* in harvested grape bunches demonstrated a reduction in disease severity with treatments compared to UTC after 10 field sprayings throughout the growing season (Figure 5b). MG exhibited an intermediate response, which was not significantly different from UTC, while TNR, ASM, and SUL/Bs showed infection percentages of 60.7%, 57.0%, and 51.5%, respectively, being significatively different from UTC under field conditions.

## 3. Discussion

The present study explored a new alternative for the sustainable control of fungal diseases in grapevine management, evaluating the efficacy of *P. protegens* strains and their formulations to induce defense genes and their effect in controlling the grapevine pathogens *E. necator* and *B. cinerea* under field conditions. These findings align with current efforts to promote integrated disease management approaches that preserve fruit quality, vineyard productivity, and environmental health [14,17,18].

The transcriptional responses measured in foliar tissue and berries provide mechanistic insight into plant–pathogen interactions. Treatments elicited gene- and time-dependent induction of marker genes: ASM induced delayed but strong *pr2* activation on leaves and early *pr1* activation on bunches, while MG and TNR induced earlier moderate responses of *pr1* and *pr2* on bunches. These gene expression patterns, which are associated with the salicylic acid (SA)-dependent pathway, are in accordance with the ISR paradigm, in which beneficial microbial treatments prime plant defense pathways, resulting in faster or stronger transcriptional responses upon challenge rather than constitutive defense activation of defenses [31]. The transient peaks observed for *pr*10, *pal* and JA-related *lox9* are consistent with energetically costly, rapidly deployed responses that return toward baseline associated with a phenomenon of defense priming [31]. However, this pattern contrasts with the more pronounced and dynamic responses observed for defense-related genes (*pr1* and *pr2*), suggesting that these three markers may contribute to a basal defense response rather than driving strong inducible activation. These molecular signatures, together with the observed reductions in disease severity, are consistent with a role of *P. protegens* and its formulations in modulating host defense responses [51]. While these gene expressions findings may suggest a potential contribution of *P. protegens* to induce defense mechanism, the variation in response could be influenced by cultivar-specific factors [52]. However, no direct comparisons among cultivars were performed in this research; therefore, such effects remain to be elucidated.

Complementary evidence from recent grapevine studies supports a dual, organ- and pathway-specific model for the defenses reported in the present study. Shen et al. [53] demonstrated that resistant fruits accumulate flavonoids (e.g., rutin, luteolin-7-glucuronide) with direct antifungal activity against *B. cinerea*, highlighting a role for metabolite-mediated fruit protection [53]. Conversely, Yan et al. [54] identified a VqMAPK3-VqERF1B module, involving a phosphorylated mitogen-activated protein kinase that synergistically activates *pr*1/*pr*5/*pr*10 transcription, along with reactive oxygen species (ROS) production, to restrict *E. necator*, underscoring a MAPK-ERF-PR regulatory axis in foliar immunity. These findings suggest that *P. protegens*-triggered transcriptional priming may act in concert with organ-specific metabolites to determine the cultivar-dependent disease outcomes observed.

The strong development of powdery mildew observed in the untreated controls, with an infection level of 74% in the leaf disc assays, confirms the well-documented susceptibility of the Chardonnay cultivar to this disease [55]. However, this method did not allow clear discrimination among resistance inducers based on *P. protegens* and ASM, although the concentrated suspension formulation MG showed a marginal reduction in disease symptoms on leaf discs, consistent with earlier reports on the biocontrol potential of this bacterium [19,56]. A similar pattern was observed in leaf discs inoculated with *B. cinerea*; however, in this case, MG resulted in a significant reduction in fungal growth. Notably, the results obtained from the in vitro leaf disc assays contrasted with those observed in the grapevine plants under field conditions, where clear differences among treatments and the control were detected. These discrepancies between in vitro and field results underline the well-known limitations of detached-tissue assays, including restricted assay duration, constrained microbial colonization, and simplified host-microbe-pathogen interactions, which can reduce statistical significance and hide field-relevant effects [47,57,58].

Field assays demonstrated that repeated applications of *P. protegens* live cells and their commercial formulations, as well as of the chemical inducer ASM, moderately reduced powdery mildew severity in both leaves and bunches. However, the magnitude of reduction achieved by *P. protegens* was generally lower than that obtained with sulfur/*Bacillus subtilis* QST-713 and the synthetic fungicide Switch^®^, indicating that these microbial treatments should be considered as complementary tools rather than stand-alone alternatives to conventional fungicides under conditions of high disease pressure. It has been reported that ASM can reduce Botrytis bunch rot incidence in grapevine cultivars, with reductions of up to 75% relative to untreated controls [59,60]. However, despite its proven efficacy against *B. cinerea*, ASM has not yet been systematically evaluated for the control of powdery mildew.

The efficacy of *P. protegens* in field experiments suggests a possible dual-activity mode of action described for many *Pseudomonas* spp., which should be based on the synthesis of antifungal metabolites (e.g., 2,4-DAPG, pyoluteorin, pyrrolnitrin) coupled with the ability to prime or trigger induced systemic resistance [19,61,62]. No statistically significant differences were observed between treatments using bacterial cells of the strains and those employing their formulated products, suggesting that formulation processes preserved the bioactivity and efficacy of the bacterial agents. In contrast, research in microbial biocontrol has emphasized that achieving consistent field performance with *Pseudomonas* formulations typically requires consideration of formulation stability, dosage, and application timing [18,63]. These factors may explain why repeated field applications in our trials resulted in improved disease suppression relative to the short exposure periods used in the in vitro leaf disc assay. Recent reviews have further emphasized the broad spectrum of mechanisms employed by *Pseudomonas* spp., ranging from direct competition and volatile emission to type VI secretion systems and the activation of plant systemic resistance [18,23].

The conventional anilinopyrimidine fungicide combination cyprodinil/fludioxonil, as expected, provided the highest level of protection against *B. cinerea* in berry bioassays. Likewise, the traditional inorganic contact fungicide sulfur, a multi-site contact product, offered rapid and reliable disease suppression under high infection pressure in the field experiment, which received six applications of this compound and four applications of *B. subtilis* QST-713 [59,64,65].

The modulation of defense gene expression, together with the partial suppression of fungal infection observed in treated Chardonnay vines, supports the potential of this approach. These results indicate that *P. protegens* formulations can activate grapevine defense pathways and contribute to disease mitigation under both controlled and field conditions, although their performance remains below that of standard fungicide programs. Although short-term leaf disc assays did not yield consistent statistically significant differences, these results likely reflect the limitations inherent to detached-tissue systems, in which altered metabolism and accelerated tissue senescence may constrain defense gene expression and microbial persistence. Consequently, the efficacy of bioinducers should ideally be validated through whole-plant and long-term field trials that more accurately reflect natural physiological conditions [66]. Nevertheless, the field experiments reported in the present study provided encouraging results, confirming the disease-suppressive capacity of *P. protegens* under vineyard conditions. However, broader temporal and spatial replication, through multi-season and multi-site trials, will be essential to confirm the stability of these effects across diverse environments and infection pressures [67]. Recent studies on grapevine immunity have identified hundreds of differentially expressed genes (DEGs) in response to powdery mildew, downy mildew, and trunk disease infections, highlighting the molecular complexity of host–pathogen interactions [68,69,70]. In this context, our analysis focused on five key genes representing the main defense pathways activated by *P. protegens* inoculation. While this targeted approach effectively captured the principal dynamics of induced resistance, it may not encompass the full scope of hormonal crosstalk, protein activity, and metabolic adjustments that contribute to durable protection [22]. Finally, formulation parameters, including microbial viability, shelf life, adjuvant composition, and *in planta* colonization behavior, remain critical determinants of field efficacy and commercial scalability [18,23]. Although these aspects were not exhaustively addressed here, they represent promising avenues for future optimization of *P. protegens*-based biocontrol products.

Overall, the results of gene expression analyses and field efficacy assessments for the control of powdery mildew and Botrytis bunch rot support the potential use of *P. protegens*-based formulations as components of integrated grapevine disease management. This approach recognizes that these bioinducers elicit defense pathways that differ in timing and durability from those triggered by classical chemical inducers such as ASM. Their use could serve as a complementary tool to inorganic contact fungicides, such as sulfur, or synthetic fungicides under field conditions. Translating these findings into robust agronomic recommendations will require factorial, multi-season field trials, along with further improvements in formulation and application conditions. Further studies should also incorporate expanded molecular profiling, including hormone quantification, metabolomics, and proteomics, to link expression peaks to functional resistance outcomes. Addressing these knowledge gaps will help clarify where and how *P. protegens* formulations can most reliably complement sustainable phytosanitary strategies in viticulture [19,31,63].

## 4. Materials and Methods

### 4.1. Plant Material

Tissue material for laboratory experiments and field trials was obtained from a *Vitis vinifera* cv. Chardonnay vineyard established in El Nogal Experimental Station at the Faculty of Agronomy of the Universidad de Concepción, Chillán, Ñuble Region, Chile (36°35′58″ S 72°04′58″ W). The vineyard was 15 years old and established on an Andisol with sandy loam texture (Arrayan series [71]), under a warm temperate Mediterranean climate characterized by dry and rainy seasons. Vines were planted at a density of 3300 plants ha^−1^, trained under a double-cordon training system, and managed with drip irrigation.

### 4.2. Microbiological Material

*P. protegens* bacteria strains Ca2, Ca6, and ChC7 isolated from wheat roots in Chilean soils were used [72]. Additionally, two biological stimulants based on these native strains of *P. protegens* were used in commercial doses: TANIRI^®^ WP (Bio Insumos Nativa^®^, Maule, Chile), a wettable powder formulation based on Ca2 and ChC7 strains (5 × 10^3^ colony-forming unit (cfu) g^−1^), and MaxGrowth (Bioprotegens Innovations SpA., Chillán, Chile), a liquid concentrated suspension based on Ca6 strain (10^8^ cfu·L^−1^) (Table 1). The bacterial strains were incubated in King’s B (KB) broth composed of peptone protease (20 g), anhydrous K_2_HPO_4_ (1.965 g), MgSO_4_ 7H_2_O (1.5 g), glycerol (10 mL), and distilled water to a final volume of 1 L, at 25 ± 0.5 °C for 48 h with constant agitation at 150 rpm [43]. Before use, bacterial cells were washed twice with a saline solution (NaCl 0.89% (*w*/*v*)).

*Botrytis cinerea* (Bc) pathogenic strain F003 was provided by the Phytopathology Laboratory of the Faculty of Agronomy at the Universidad de Concepción, Chillán, Ñuble Region, Chile. The strain was originally isolated from grape berries and characterized as highly pathogenic on grapevine. Fungal identification was confirmed by sequencing the internal transcribed spacer (ITS) region through capillary electrophoresis sequencing (CES; Macrogen Inc., Seoul, Republic of Korea) (Table A3) and by using specific *B. cinerea* primers based on IGS spacer (Bc3 primers [73]). The pathogen was grown on potato dextrose agar plates (PDA; Difco Inc., New York, NY, USA) at 24 °C for 10 days. Conidial suspensions were prepared by flooding culture plates with sterile distilled water (SDW) containing 0.05% (*v*/*v*) Tween-80, followed by gentle scraping of the agar surface with a sterile scalpel. The suspension was filtered through two layers of sterilized gauze previously autoclaved at 120 °C for 15 min. Conidia concentration was determined using a hemocytometer (Hirschmann^®^, Eberstadt, Germany) under a light optical microscope, and the suspension was diluted with SDW to a final concentration of 10^6^ conidia mL^−1^ for subsequent bioassays [48].

Fresh conidia of *Erysiphe necator* were obtained from naturally infected plants from the vineyard described above and used in the laboratory bioassays. Spores were gently collected from infected leaves using a soft brush, following the method described by Lukšić et al. [47].

### 4.3. Relative Expression Assays to Determine Defense Gene Induction

#### 4.3.1. RNA Extraction and cDNA Synthesis

A field assay was conducted in the experimental Chardonnay vineyard to evaluate the expression of five defense-related genes. Plots consisting of five grapevine plants were arranged in a randomized block design. Treatments were applied using a mist blower backpack sprayer (Stihl^®^ SR 450, Waiblingen, Germany) until runoff. The evaluated treatments included two bioproduct formulations based on *P. protegens* (MG and TNR), a chemical resistance inducer formulated as water-soluble granules containing acibenzolar-S-methyl (ASM) at a concentration of 500 mg g^−1^ as the active ingredient (BION^®^ 50 WG; Syngenta^®^, Basel, Switzerland), and an untreated control (UTC; Table 2). Each treatment was replicated six times.

Four leaves per plant at the EL 27 phenological stage [74] were collected from the central plant of each plot at 24 h, 7 and 14 days after treatment application. Samples were immediately frozen in liquid nitrogen. Additionally, 12 berries from four bunches at the EL 35 stage were collected from the central plant of each plot at 7, 14, and 21 days after treatment (DAT) application. Sampled material was stored at −80° C in an ultra-low-temperature freezer until further analysis.

Two hundred milligrams (200 mg) of frozen leaf or berry tissue were extracted by using an RNA extraction kit, namely the Spectrum^TM^ Plant Total RNA Kit (Sigma-Aldrich^®^, St. Louis, MO, USA), according to the manufacturer’s instructions [20]. RNA integrity was visualized in 1% agarose gels, using the molecular weight marker GeneRuler^TM^ 1 kb Plus DNA Ladder (Thermo Scientific^TM^, Waltham, MA, USA). RNA concentration and purity were determined using an Epoch^®^ spectrophotometer (BioTek Instruments, Winooski, VT, USA). The samples were stored at −80 °C until complementary DNA (cDNA) synthesis.

Residual genomic DNA was removed by digestion with DNase I RNase-free (Thermo Scientific^TM^, Waltham, MA, USA), and then cDNA was synthesized using the High-Capacity cDNA kit (Applied Biosystems^TM^, Foster City, CA, USA) according to the manufacturer’s instructions, with the inclusion of RNase inhibitor (Thermo Scientific^TM^, Waltham, MA, USA). Digestion and cDNA synthesis were carried out using a MaxyGene^TM^ II thermal cycler (Axygen^®^, Union City, CA, USA).

#### 4.3.2. Studied Genes, Primer Validation, and qPCR Quantification

The expression levels of genes encoding pathogenesis-related protein 1 (*pr1*), β-1,3-glucanase (*pr2*), pathogenesis-related protein 1 (*pr10*), phenylalanine ammonia-lyase (*pal*), and lipoxygenase 9 (*lox9*) were analyzed (Table 3). The ubiquitin (*ubc*) and actin (*act*) genes were used as internal reference genes. The expression stability of *act* and *ubc* under the experimental conditions employed in this study was previously validated by Toth et al. [75] and Borges et al. [76], respectively.

All primers were previously validated in silico using Primer-BLAST (NCBI; Primer3 v. 2.5.0) and experimentally by endpoint PCR. Endpoint PCR amplifications for each primer were performed in a MaxyGene^TM^ II thermal cycler (Axygen^®^) using the following program: an initial step of 95 °C for 5 min, followed by 35 cycles of 94 °C for 30 s, 60 °C for 30 s and 72 °C for 30 s, with a final step of 72 °C for 10 min and a hold at 4 °C until sample removal. PCR products were resolved at 1% (*w*/*v*) agarose gels prepared with 0.5x TBE Buffer and visualized using SYBR^®^ Safe DNA Gel Stain (Invitrogen, Thermo Scientific^TM^, Waltham, MA, USA). For each reaction, 5 µL of PCR product was mixed with 1 µL of 6X DNA Loading Dye buffer (Thermo Scientific^TM^, Waltham, MA, USA) and loaded onto the gel. A GeneRuler^TM^ 100 bp DNA Ladder (Thermo Scientific^TM^, Waltham, MA, USA) was used as a molecular weight marker. Primer efficiency was subsequently assessed by qPCR quantification through amplification curves using cDNA.

The relative expression was quantified using the 2^−ΔΔCt^ method [77] with the StepOnePlus^TM^ Real-Time PCR System (Applied Biosystems^TM^, Foster City, CA, USA). Each reaction contained a mixture of 5 μL of KAPA SYBR^®^ FAST qPCR mix (Roche, Mannheim, Germany), 10 ng of cDNA, 0.5 μM of each primer, and molecular-grade water (nuclease-free) to a final volume of 10 μL. The qPCR plate was loaded in three technical replicates of each sample. Gene expression levels in the treated plants were quantified relative to the untreated controls (UTC) and normalized against two reference housekeeping genes.

Data analysis was conducted with StepOne Software v2.3. Statistical analyses were carried out using analysis of variance (ANOVA) on plot means, followed by Tukey’s Honestly Significant Difference (HSD) test (*p* ≤ 0.05), implemented in R software v4.1.1 (https://www.r-project.org/; accessed on 13 January 2025). Prior to ANOVA, data were tested for normality and homoscedasticity; when required, percentage data were subjected to arcsine-square root transformation.

### 4.4. Leaf Disc Control Assays to Assess the Suppression of Botrytis cinerea and Erysiphe necator in Grapevine Induced by Pseudomonas protegens

The experiment was organized in a completely randomized design with four replicates per treatment. In each replicate, three Chardonnay grapevine plants were sprayed with the treatments described in Table 4, resulting in a total of 12 plants per treatment. Treatments were applied at the EL 31 phenological stage (BBCH 75; [50]) using a mist blower backpack sprayer (Stihl^®^ SR 450), spraying until runoff.

Seven days after treatment application, six healthy, fully developed leaves were sampled per treatment from each plot (experimental unit) and transported to the laboratory. From each leaf two leaf discs (two subsamples) were excised using a 12 mm cork borer. Individual leaf discs from each treatment were placed in group of six on 1% (*w*/*v*) water-agar (Bacto™ Agar; BD Difco, Franklin Lakes, NJ, USA) in plastic Petri dishes [20]. Each treatment had four replicates (Table 4).

Each disc from each replicate was inoculated with an aliquot of 10 μL of a *B. cinerea* F003 conidial suspension (10^6^ conidia mL^−1^) and incubated at 24 °C for seven days post-inoculation (DPI) under dark conditions. At this time point, leaf discs were evaluated for mycelial growth and necrotic symptoms. A separate set of leaf discs was inoculated with fresh conidia *E. necator* obtained from naturally infected leaves. The plates were maintained for seven days in a climate chamber at 25 °C and 80% relative humidity under a 16 h light/8 h dark photoperiod. In both experiments, a set of non-inoculated leaf discs obtained from the untreated control (UTC) sprayed with SDW was included. Disease severity in both bioassays were assessed by measuring the leaf area covered by fungal structures and necrotic lesions induced by the pathogens [47].

### 4.5. In Vivo Assessment of the Efficacy of Microbial and Chemical Resistance Inducers on Grape Berries Inoculated by Botrytis cinerea

Two bioassays were conducted to determine the level of control on Botrytis bunch rot observed in grapevines previously treated under field conditions with *P. protegens* strains, their formulations, and ASM (Table 5).

In the first bioassay, a field experiment was conducted in the experimental vineyard, where healthy grapes bunches showed low disease pressure. The treatments described in Table 5 were sprayed directly onto the bunches of three plants per plot, seven days before harvest. Treatments were arranged in a randomized block design with four replicates. From the central plant of each treated plot, four bunches per vine were harvested and transported to the laboratory in a cooler with disinfected ice packs. Bunches were surface-disinfected with 1.5% (*w*/*v*) sodium hypochlorite, followed by two washes with SDW to remove epiphytic microflora. Six healthy berries were selected from each bunch (24 berries per plot), carefully cut from the rachis with the pedicel intact, and placed in an individual humid chamber, which contained the assessed seven treatments. Four humid chambers per plot were assessed to obtain the plot mean. Each berry was individually inoculated with a 10 µL aliquot of *B. cinerea* F003 conidial suspension (10^6^ conidia mL^−1^).

In the second bioassay, healthy, untreated bunches were collected from an area of the experimental Chardonnay vineyard that had not been treated with any biological or microbial pesticides. From these bunches, berries were excised from the rachis with the pedicel intact and sprayed with the treatments described in Table 5. Treated berries were allowed to dry at room temperature and subsequently inoculated with the pathogen aliquot and conidial suspension described above. Six berries per treatment plot were carefully placed in humidity chambers [48]. Berries sprayed with SDW and then inoculated with the conidial suspension were used as an untreated control (UTC).

In both assays, each humidity chamber was prepared with six berries per treatment and was maintained at 21 ± 1 °C and 95 to 100% relative humidity for 5 DPI. The disease severity index (DSI) was visually assessed according to the following scale [48]: 0 = no infection, 1 = tiny spot, 2 = one infected spot, 3 = two to four infected spots, 4 ≤ 50% of the berry surface infected with typical sporulation, and 5 ≥ 50% of the berry surface infected with typical sporulation. DSI was calculated according to the following formula: DSI (%) = [Σ(class frequency × score of rating class)]/[(total number of evaluated leaves) × (maximal disease index)] × 100 (1) [78]. DSI was analyzed with ANOVA on plot means, followed by Tukey’s HSD test (*p* ≤ 0.05) to determine significant differences among the treatments.

### 4.6. Efficacy Assay of Pseudomonas protegens to Control Gray Mold and Powdery Mildew in Chardonnay Grapevines Under Field Conditions

A field experiment was conducted during the 2023–2024 growing season to assess the treatments described in Table 6. Each experimental unit consisted of five grapevine plants per plot, arranged in a randomized block design with six replicates per treatment. Each treatment was sprayed ten times throughout the growing season. Applications were conducted at 14-day intervals, starting when shoots reached 10–15 cm in length and continuing until two weeks before harvest. Treatments were applied with a mist-blower backpack sprayer (Stihl^®^ SR 450) until runoff. The central three plants of each experimental unit were used for the assessment of gray mold and powdery mildew infection.

A commercial control program (SUL/Bs) was implemented, consisting of six applications of sulfur 72% *w*/*v* (Acoidal^®^ Flo, Quimetal Industrial S.A., Santiago, Chile) at a concentration of 2 mL·L^−1^, followed by four applications of *Bacillus subtilis* QST-713 (Serenade^®^ ASO, Bayer, Leverkusen, Germany) applied at a concentration of 6 mL·L^−1^.

#### 4.6.1. Assessment of Powdery Mildew Under Field Conditions

The overall health status of each plant was evaluated at the EL 31 stage (BBCH 75; [50]). Powdery mildew symptoms (white to grayish mycelium) were recorded on leaves (adaxial surfaces) and bunches. Leaf DSI was visually evaluated after four treatment applications at the EL 31 stage (BBCH 75), using a modified scale adapted from the International Plant Genetic Resources Institute (IPGRI): 0 = absent; 1 = fewer than five small spots; 2 = five to twenty growing spots; 3 = widespread powdery mildew with dense sporulation, as described by Barba et al. [50]. For each experimental plot, fifty leaves per plant were randomly selected from the three central plants to minimize edge effects, resulting in a total of 150 leaves evaluated per plot. The disease severity index (DSI) was calculated as described above.

Powdery mildew severity on bunches was evaluated by determining the percentage of the bunch exhibiting pathogen symptoms. Ten grape bunches were evaluated from each of the three central vines per experimental unit across six replicates, resulting in a total of 30 bunches per plot.

#### 4.6.2. Assessment Botrytis Bunch Rot Control Under Field Conditions

Bunches from the three central vines of each plot were harvested at the EL 38 phenological stage; BBCH 89 (19–20 °Brix) after 10 applications of the treatments during the season. Bunches were transported to the laboratory and placed into humidity chambers (Hermetic plastic boxes; 32 × 21 × 6 cm) in groups of 6, totaling 18 bunches per plot. Bunches were maintained at 24 ± 1 °C and 95 to 100% relative humidity for five days. Rot disease severity was then evaluated through visual inspection of the percentage of the bunch area showing signs and symptoms. Data were analyzed using ANOVA on plot means, followed by Tukey’s HSD test (*p* ≤ 0.05), to determine significant differences among the treatments. All statistical analyses conducted for laboratory and field experiments were performed using R software (v4.1.1).

## 5. Conclusions

This study demonstrates that formulations based on *Pseudomonas protegens* are capable of activating grapevine defense responses, as evidenced by the upregulation of *pr1* and *pr2*, which are established markers of the salicylic acid (SA)-dependent pathway. In contrast, *lox*9, associated with the JA-related oxylipin pathway, and *pal*, associated with the phenylpropanoid metabolism genes pathway, exhibited less stable modulation and tissue-dependent expression patterns, with no consistent induction observed in berries under microbial treatments. Bacterial treatments contributed to reducing the severity of *Erysiphe necator* and *Botrytis cinerea* under field conditions, although the strongest disease suppression was obtained with sulfur/*Bacillus subtilis* QST-713 and synthetic fungicide treatments. Overall, *P. protegens* formulations may contribute as a complementary, more sustainable component within integrated disease management programs in viticulture; however, their efficacy under the conditions evaluated was limited and remained inferior to those of sulfur and the conventional synthetic fungicide program. Further studies should focus on elucidating the molecular mechanisms underlying induced resistance and on the long-term effects of bacterial formulations in vineyard agroecosystems.

## Figures and Tables

**Table 1:** Overview of biological treatments based on native *Pseudomonas protegens* strains used throughout the study. The table standardizes treatment codes, strain composition, formulation type, application rates, and experimental contexts, including gene expression assays, leaf disc assays, berry assays, and field evaluations of powdery mildew (PM) and Botrytis bunch rot (BBR).

Treatment Code	Description/Commercial Name	*P. protegens* Strain (s)	Formulation	Applied Dose	Experimental Context of Use
PP1	*P. protegens* Ca2 and ChC7 (laboratory strain)	Ca2 + ChC7	Liquid bacterial suspension	10^6^ cfu·L^−1^	Leaf disc assays; berry assays
PP2	*P. protegens* Ca6 (laboratory strain)	Ca6	Liquid bacterial suspension	10^6^ cfu·L^−1^	Berry assays
TNR	TANIRI^®^ WP (Bio Insumos Nativa^®^, Maule, Chile)	Ca2 + ChC7	Wettable powder (WP)	1 g·L^−1^ (5 × 10^3^ cfu g^−1^)	Gene expression assays; leaf disc assays; berry assays; field PM-BBR assays
MG	MaxGrowth (Bioprotegens Innovations SpA., Chillán, Chile)	Ca6	Liquid concentrated suspension	1 mL·L^−1^ (10^8^ cfu·L^−1^)	Gene expression assays; leaf disc assays; berry assays; field PM-BBR assays

**Table 2:** Treatments evaluated for the assessment of defense gene induction in grapevine cv. Chardonnay following field application of *Pseudomonas protegens*-based bioproduct formulations and a chemical resistance inducer under field conditions.

Code	Treatment	Active Agent	Concentration
UTC	Water	-	-
MG	MaxGrowth (Bioprotegens Innovations SpA.)	*P. protegens* Ca6 strain	1 mL·L^−1^
TNR	TANIRI^®^ WP (Bio Insumos Nativa^®^)	*P. protegens* Ca2 and ChC7 strains	1 g·L^−1^
ASM	BION^®^ 50 WG (Syngenta^®^)	Acibenzolar-S-methyl	0.2 g·L^−1^

**Table 3:** Grapevine genes analyzed, forward and reverse primer sequences, amplicon length (bp), and supporting references used for grapevine gene expression analyses.

Gene	Forward Primer (5′-3′)	Reverse Primer (5′-3′)	Amplicon Length (bp)	Reference
Actin (*act*)	CCCCACCTCAACACATCTCC	TCCATTGTCCACAGGAAGTGC	138	Toth et al. [75]
Ubiquitin (*ubc*)	CACCCGAATATAAACAGCAATGG	ACAGCAACACCTTGGAGATAG	161	Borges et al. [76]
Pathogenesis-related protein 1 (*pr1*)	TGGCTACCTACGCCCAGAAC	CGGTGCCTGTCAATGAAG	117	Toth et al. [75]
Β-1,3-glucanase (*pr2*)	TCAATGGCTGCAATGGTGC	CGGTCGATGTTGCGAGATTTA	155	Lakkis et al. [20]
Phenylalanine ammonia-lyase (*pal*)	TCCTCCCGGAAAACAGCTG	TCCTCCAAATGCCTCAAATCA	101	Lakkis et al. [20]
Lipoxygenase 9 (*lox9*)	CCCTTCTTGGCATCTCCCTTA	TGTTGTGTCCAGGGTCCATTC	101	Lakkis et al. [20]
Pathogenesis-related protein 10 (*pr10*)	GTTTTGACTGACGGCGTTGA	TGGTGTGGTACTTGCTGGTGTT	99	Monteiro et al. [52]

**Table 4:** Treatments and concentrations applied in the field to evaluate the suppression of *Erysiphe necator* and *Botrytis cinerea* in grapevine leaf discs under laboratory conditions. Treatments included biological control agents, commercial bioproducts, and a chemical resistance inducer.

Code	Treatments	Concentration
UTC	Untreated control (Water)	-
PP1	*Pseudomonas protegens* Ca2 and ChC7	10^6^ cfu·L^−1^
TNR	TANIRI^®^ WP (Bio Insumos Nativa^®^)	1 g·L^−1^
MG	MaxGrowth (Bioprotegens Innovations SpA.)	1 mL·L^−1^
ASM	BION^®^ 50 WG (Syngenta^®^)	0.2 g·L^−1^

**Table 5:** Treatments and concentrations evaluated to determine the efficacy of microbial and chemical resistance inducers on berries inoculated by *Botrytis cinerea*. Treatments included a negative control (sterile distilled water; SDW), bacterial suspensions of *Pseudomonas protegens* strains, commercial bioproducts based on these bacterial strains, a chemical plant defense inducer, and a synthetic fungicide. Concentrations are expressed as colony-forming units (CFUs) L^−1^ or product dosage per liter, according to manufacturers’ recommendations.

Code	Treatments	Concentration
UTC	Untreated control (SDW)	-
PP1	*P. protegens* Ca2 and ChC7 strains	10^6^ cfu·L^−1^
PP2	*P. protegens* Ca6 strain	10^6^ cfu·L^−1^
MG	MaxGrowth (Bioprotegens Innovations SpA.)	1 mL·L^−1^
TNR	TANIRI^®^ WP (Bio Insumos Nativa^®^)	1 g·L^−1^
ASM	BION^®^ 50 WG (Syngenta^®^)	0.2 g·L^−1^
SWT ^1^	Switch^®^ 62.5 WG (Syngenta^®^)	1 g·L^−1^

^1^ Water-soluble granules formulation composed of cyprodinil (375 mg g^−1^) and fludioxonil (250 mg g^−1^) as active components.

**Table 6:** Treatments and concentrations evaluated to determine the efficacy of microbial and chemical resistance inducers for the control of gray mold and powdery mildew in Chardonnay grapevines under field conditions. Treatments included a negative control, two commercial bioproducts based on *Pseudomonas protegens* (MG and TNR) strains, a chemical plant defense inducer (ASM), and an inorganic and a biological fungicide-based commercial control program based in six sprayings of sulfur and four of *Bacillus subtilis* QST-713 (SUL/Bs). The applied concentration or dose for each treatment is indicated.

Code	Treatment	Active Agent	Dose
UTC	Sterile distilled water (SDW)	-	-
MG	MaxGrowth (Bioprotegens Innovations SpA.)	Ca6 Strain	1 mL·L^−1^
TNR	TANIRI^®^ WP (Bio Insumos Nativa^®^)	Ca2 and ChC7 Strains	1 g·L^−1^
ASM	BION^®^ 50 WG (Syngenta^®^)	Acibenzolar-S-methyl (ASM)	0.2 g·L^−1^
SUL/Bs	Acoidal^®^ Flo/Serenade^®^ ASO	Sulfur 72%/*B. subtilis* QST-713 1.4%	2 mL·L^−1^/6 mL·L^−1^

## Data Availability

The raw data supporting the conclusions of this article are available in the Supplementary Materials.

## Supplementary Materials

The following supporting information can be downloaded at: https://www.mdpi.com/article/10.3390/plants15091371/s1, Table S1, Severity of *Erysiphe necator* on leaf discs and severity of *Botrytis cinerea* infection on leaf discs, measured as the percentage of leaf surface area affected. The dataset corresponds to the mean values of observations per replicate. Treatments included control treated with distilled water control (SDW; UTC), *Pseudomonas protegens* strains Ca2 and ChC7 (PP1), a dust formulation (TANIRI^®^ WP [TNR]), a liquid suspension formulation (MaxGrowth [MG]), and acibenzolar-S-methyl (BION^®^ 50 WG [ASM]). Leaf disks were assessed 7 days after inoculation and maintained at 25 °C; Table S2, Severity of *Erysiphe necator* on leaf discs, measured as the percentage of leaf surface area affected. The dataset corresponds to the mean values of observations per treatments. Standard error (SE); Table S3, Severity of *Botrytis cinerea* infection on leaf discs, measured as the percentage of leaf surface area affected. The dataset corresponds to the mean values of observations per treatments. Statistical groupings in panel b are indicated by different letters above the bars, based on ANOVA followed by Tukey’s HSD test (*p* ≤ 0.05); Table S4, Disease severity index (DSI) of *Botrytis cinerea* (Bc) infection in grape berries treated with *Pseudomonas protegens* strains and their formulations (PP1, PP2, MG, TNR), acibenzolar-S-methyl (ASM), and a cyprodinil + fludioxonil fungicide mixture (SWT). Treatments were applied in a Chardonnay vineyard seven days prior to harvest, followed by artificial inoculation with Bc under field conditions (a), or applied post-harvest and subsequently inoculated with Bc under laboratory conditions (b). Disease severity was evaluated after 5 days of incubation in humidity chambers maintained at 22 °C. Six berries were placed in each humidity chamber per treatment, and the experiment was replicated across 16 independent biological replicates per treatment (*n* = 96). The dataset corresponds to the mean values of observations per replicate; Table S5, Disease severity index (DSI) of *Botrytis cinerea* (Bc) infection in grape berries treated with *Pseudomonas protegens* strains and their formulations (PP1, PP2, MG, TNR), acibenzolar-S-methyl (ASM), and a cyprodinil + fludioxonil fungicide mixture (SWT). Treatments were applied in a Chardonnay vineyard seven days prior to harvest, followed by artificial inoculation with Bc under field conditions. Disease severity was evaluated after 5 days of incubation in humidity chambers maintained at 22 °C. Six berries were placed in each humidity chamber per treatment, and the experiment was replicated across 16 independent biological replicates per treatment (*n* = 96). The dataset corresponds to the mean values of observations per treatments. Different letters indicate significant differences according to Tukey’s HSD test (*p* ≤ 0.05); Table S6, Disease severity index (DSI) of *Botrytis cinerea* (Bc) infection in grape berries treated with *Pseudomonas protegens* strains and their formulations (PP1, PP2, MG, TNR), acibenzolar-S-methyl (ASM), and a cyprodinil + fludioxonil fungicide mixture (SWT). Treatments were applied post-harvest and subsequently inoculated with Bc under laboratory conditions. Disease severity was evaluated after 5 days of incubation in humidity chambers maintained at 22 °C. Six berries were placed in each humidity chamber per treatment, and the experiment was replicated across 16 independent biological replicates per treatment (*n* = 96). The dataset corresponds to the mean values of observations per treatments. Different letters indicate significant differences according to Tukey’s HSD test (*p* ≤ 0.05); Table S7, Powdery mildew disease severity index (DSI) on grapevine leaves evaluated at EL 31 stage (BBCH 75) after four spray treatments. Disease severity was visually assessed using the scale described by Barba et al. [50]: 0 = absent, 1 = <5 small spots, 2 = 5–20 expanding spots, 3 = widespread powdery mildew with dense sporulation. Treatments included untreated control (UTC), MaxGrowth (MG), TANIRI^®^ WP (TNR), acibenzolar-S-methyl (ASM), and sulfur 72% (SUL). Data represent mean DSI values per treatment across six replicates. Fifty leaves per grapevine were randomly selected from three central grapevines within each plot (*n* = 900). The dataset corresponds to the mean values of observations per replicate; Table S8, Powdery mildew disease severity index (DSI) on grapevine leaves evaluated at EL 31 stage (BBCH 75) after four spray treatments. Data represent mean DSI values per treatment across six replicates. Fifty leaves per grapevine were randomly selected from three central grapevines within each plot (*n* = 900). Mean comparisons were performed using ANOVA, and significant differences among treatments were identified by Tukey’s HSD test (*p* ≤ 0.05). Different letters indicate significant differences. The dataset corresponds to the mean values of observations per treatments; Table S9, Efficacy of control of natural infection of powdery mildew (PM) and Botrytis bunch rot (BBR) on Chardonnay grape bunches by biological and chemical bioinducers after 10 spraying under field conditions. Disease severity on grape bunches, expressed as the percentage of organs with visible signs or symptoms was assessed by visual inspection. Treatments included an untreated control (UTC), MaxGrowth (MG), TANIRI^®^ WP (TNR), and BION^®^ 50 WG (ASM) and Sulfur 72% (SUL). Powdery mildew severity was assessed at the EL 31 stage after four treatments application (*n* = 180), while *Botrytis cinerea* severity was assessed on harvest grape bunches (EL38) after 7 days of incubation in humidity chambers at 25 °C (*n* = 108). The dataset corresponds to the mean values of observations per replicate; Table S10, Efficacy of control of natural infection of powdery mildew (PM) on Chardonnay grape bunches by biological and chemical bioinducers after 10 spraying under field conditions. Treatments were compared by ANOVA and different letters above the bars denote significant differences among treatments according to Tukey’s HSD test (*p* ≤ 0.05). The dataset corresponds to the mean values of observations per treatments; Table S11, Efficacy of control of natural infection of Botrytis bunch rot (BBR) on Chardonnay grape bunches by biological and chemical bioinducers after 10 spraying under field conditions. Treatments were compared by ANOVA and different letters above the bars denote significant differences among treatments according to Tukey’s HSD test (*p* ≤ 0.05). The dataset corresponds to the mean values of observations per treatments.

## Abbreviations

The following abbreviations are used in this manuscript:

PM	powdery mildew
BBR	botrytis bunch rot
ASM	acibenzolar-S-methyl
SA	salicylic acid
JA	jasmonic acid
IR	induced resistance
SAR	systemic acquired resistance
WP	wettable powder
UTC	untreated control
SDW	sterile distilled water
qPCR	quantitative real-time PCR
C_t_	cycle threshold
cDNA	complementary DNA
bp	base pairs
CFU	colony-forming units
WG	water-dispersible granules
DEGs	differentially expressed genes
IPGRI	International Plant Genetic Resources Institute
BBCH	Biologische Bundesanstalt, Bundessortenamt und Chemische Industrie
EL	Eichhorn & Lorenz
DSI	disease severity index
DAT	days after treatment
DPI	days post-inoculation

## Appendix A

**Table A1:** Relative expression levels of defense-related genes in grapevine leaves. Values represent mean ± SD (*n* = 6). Different letters indicate statistically significant differences according to one-way ANOVA, followed by Tukey’s HSD test (*p* ≤ 0.05).

Treatment × Time	*pr1* (Mean ± SD)	*pr2* (Mean ± SD)	*pr10* (Mean ± SD)	*pal* (Mean ± SD)	*Lox9* (Mean ± SD)
MG-1 DAT	0.77 ± 0.45 a	0.13 ± 0.11 a	1.32 ± 1.20 b	0.28 ± 0.19 a	0.95 ± 0.92 a
MG-7 DAT	1.09 ± 0.13 ab	1.44 ± 0.75 bc	1.28 ± 0.80 bc	0.82 ± 0.19 b	1.34 ± 0.50 b
MG-14 DAT	1.19 ± 0.47 b	3.36 ± 1.37 d	0.83 ± 0.22 a	0.96 ± 0.56 b	0.98 ± 0.29 a
TNR-1 DAT	0.36 ± 0.11 a	0.12 ± 0.05 a	0.52 ± 0.05 a	0.14 ± 0.02 a	0.38 ± 0.04 a
TNR-7 DAT	1.20 ± 0.43 ab	1.77 ± 0.66 c	1.52 ± 0.42 bc	0.78 ± 0.10 b	1.09 ± 0.26 ab
TNR-14 DAT	1.66 ± 0.61 b	3.23 ± 0.01 d	0.69 ± 0.80 a	0.88 ± 1.21 ab	1.01 ± 1.60 ab
ASM-1 DAT	0.52 ± 1.77 c	0.09 ± 0.28 a	0.96 ± 1.91 b	0.18 ± 0.14 a	0.92 ± 0.65 ab
ASM-7 DAT	1.28 ± 0.59 ab	1.68 ± 1.07 c	1.77 ± 0.66 ab	0.55 ± 0.29 a	1.44 ± 0.46 b
ASM-14 DAT	1.11 ± 0.32 ab	4.81 ± 1.86 e	0.84 ± 0.21 a	0.64 ± 0.19 a	1.02 ± 0.11 ab

**Table A2:** Relative expression levels of defense-related genes in grapevine bunches. Values represent mean ± SD (*n* = 6). Different letters indicate statistically significant differences according to one-way ANOVA, followed by Tukey’s HSD test (*p* ≤ 0.05).

Treatment × Time	*pr1* (Mean ± SD)	*pr2* (Mean ± SD)	*pr10* (Mean ± SD)	*pal* (Mean ± SD)	*Lox9* (Mean ± SD)
MG-1 DAT	4.43 ± 3.38 ab	0.92 ± 0.45 c	1.85 ± 1.28 bc	0.94 ± 0.46 bc	0.60 ± 0.12 c
MG-7 DAT	1.58 ± 1.40 b	1.89 ± 2.37 bc	1.16 ± 0.63 c	0.71 ± 1.05 c	0.75 ± 0.35 bc
MG-14 DAT	0.74 ± 0.89 b	4.62 ± 8.67 ab	0.36 ± 0.23 d	0.77 ± 0.42 c	0.93 ± 1.32 bc
TNR-1 DAT	3.87 ± 3.91 ab	1.16 ± 0.77 bc	2.05 ± 1.69 ab	1.17 ± 0.70 b	0.73 ± 0.24 bc
TNR-7 DAT	3.57 ± 2.90 ab	3.52 ± 3.70 ab	1.94 ± 1.55 bc	0.94 ± 1.12 bc	0.71 ± 0.19 bc
TNR-14 DAT	1.49 ± 1.74 b	1.23 ± 2.25 bc	0.40 ± 0.28 d	0.90 ± 0.68 bc	0.83 ± 1.33 bc
ASM-1 DAT	5.40 ± 1.94 a	1.40 ± 0.82 bc	2.58 ± 0.79 a	1.35 ± 0.71 a	1.36 ± 0.37 a
ASM-7 DAT	2.24 ± 2.89 ab	1.88 ± 1.41 bc	1.42 ± 0.76 bc	0.37 ± 0.30 d	0.72 ± 0.43 bc
ASM-14 DAT	2.74 ± 3.00 ab	0.97 ± 1.46 c	0.67 ± 0.56 cd	0.43 ± 0.27 d	0.38 ± 0.32 c

**Table A3:** BLASTn results of *Botrytis cinerea* F003 isolate, obtained through capillary electrophoresis sequencing of the ITS and RPB2 regions. The table summarizes the top hits against the NCBI database, including scientific name, maximum and total alignment scores, query coverage, E-values, percentage identity, accession length, and accession number. Both markers consistently identified the isolate as *Botrytis cinerea*, with 100% identity for ITS (Accession MT293592.1) and 99.87% identity for RPB2 (Accession XM_024697031.1), confirming species-level resolution.

Zone	Scientific Name	Max Score	Total Score	Query Cover	E Value	Per. Ident (%)	Acc. Len	Accession
ITS	*Botrytis cinerea*	628	628	1	1 × 10^−175^	100.0	426	MT293592.1
RPB2	*Botrytis cinerea*	1439	1439	1	0.0	99.9	4394	XM_024697031.1
