# Supplementary material for: Induction of Defense Responses and Partial Control of Powdery Mildew and Gray Mold in Vitis vinifera cv. Chardonnay by Pseudomonas protegens-Based Formulations
Source: Plants (Basel). 2026 Apr 30;15(9):1371. doi: 10.3390/plants15091371 (PMC13164928; doi:10.3390/plants15091371)
Supplement: Supplementary file 1 [file plants-15-01371-s001.zip › plants-4232808-supplementary.pdf]

Supplementary Material: additional tables manuscript plants 4232808

Table S1: Severity of *Erysiphe necator* on leaf discs and severity of *Botrytis cinerea* infection on leaf discs, measured as the percentage of leaf surface area affected. The dataset corresponds to the mean values of observations per replicate. Treatments included control treated with distilled water control (SDW; UTC), *Pseudomonas protegens* strains Ca2 and ChC7 (PP1), a dust formulation (TANIRI® WP [TNR]), a liquid suspension formulation (MaxGrowth [MG]), and acibenzolar-S-methyl (BION® 50 WG [ASM]). Leaf disks were assessed 7 days after inoculation and maintained at 25 °C.

Treatment	*Erysiphe necator*	*Botrytis cinerea*
UTC - R1	68.75	73.75
UTC - R2	81.81	90.00
UTC - R3	72.50	73.75
UTC - R4	72.92	93.33
PP1 - R1	77.08	85.00
PP1 - R2	84.58	91.67
PP1 - R3	43.75	73.75
PP1 - R4	72.50	86.67
TNR - R1	77.64	75.00
TNR - R2	77.50	88.33
TNR - R3	43.75	85.00
TNR - R4	76.25	81.67
MG - R1	75.00	56.25
MG - R2	62.64	66.67
MG - R3	35.00	80.00
MG - R4	67.92	66.67
ASM - R1	50.97	88.75
ASM - R2	81.53	80.00
ASM - R3	75.00	62.50
ASM - R4	66.25	75.00

Table S2: Severity of *Erysiphe necator* on leaf discs, measured as the percentage of leaf surface area affected. The dataset corresponds to the mean values of observations per treatments. Standard error (SE).

Treatment	*Erysiphe necator* severity mean	SE
UTC	73.99	2.77
PP1	69.48	8.93
TNR	68.78	8.35
MG	60.14	8.75
ASM	68.44	6.61

Table S3: Severity of *Botrytis cinerea* infection on leaf discs, measured as the percentage of leaf surface area affected. The dataset corresponds to the mean values of observations per treatments. Statistical groupings in panel b are indicated by different letters above the bars, based on ANOVA followed by Tukey's HSD test ($p \leq 0.05$).

Treatment	*Botrytis cinerea* severity mean	SE	Tukey's HSD test ($p \leq 0.05$).
UTC	82.71	5.22	a
PP1	84.27	3.78	a
TNR	82.50	7.32	a
MG	67.40	4.87	b
ASM	76.56	5.48	ab

Table S4: Disease severity index (DSI) of *Botrytis cinerea* (Bc) infection in grape berries treated with *Pseudomonas protegens* strains and their formulations (PP1, PP2, MG, TNR), acibenzolar-S-methyl (ASM), and a cyprodinil + fludioxonil fungicide mixture (SWT). Treatments were applied in a Chardonnay vineyard seven days prior to harvest, followed by artificial inoculation with Bc under field conditions (a), or applied post-harvest and subsequently inoculated with Bc under laboratory conditions (b). Disease severity was evaluated after 5 days of incubation in humidity chambers maintained at 22 °C. Six berries were placed in each humidity chamber per treatment, and the experiment was replicated across 16 independent biological replicates per treatment ($n = 96$). The dataset corresponds to the mean values of observations per replicate.

Treatment	Disease severity index of Bc *(a)*	Disease severity index of Bc *(b)*
UTC - R1	7.17	9.33
UTC - R2	5.33	5.17
UTC - R3	4.67	4.17
UTC - R4	3.67	4.00
UTC - R5	4.00	2.83
UTC - R6	3.83	2.33
PP1 - R1	3.17	2.50
PP1- R2	3.00	2.67
PP1 - R3	2.50	2.00
PP1- R4	2.33	4.67
PP1 - R5	2.83	2.83
PP1- R6	2.83	2.00
PP2 - R1	4.33	4.83
PP2 - R2	3.50	3.17
PP2 - R3	3.00	3.33
PP2 - R4	3.00	2.83
PP2 - R5	2.50	3.50
PP2 - R6	2.33	2.00
MG - R1	4.17	2.50
MG - R2	4.00	2.00
MG - R3	2.00	2.00
MG - R4	3.17	2.33

MG - R5	2.50	2.00
MG - R6	3.17	2.00
TNR - R1	3.00	4.50
TNR - R2	2.67	2.67
TNR - R3	2.33	2.83
TNR - R4	2.67	2.00
TNR - R5	2.83	2.00
TNR - R6	3.83	2.17
ASM - R1	3.67	2.50
ASM - R2	3.33	2.00
ASM - R3	2.33	3.50
ASM - R4	4.17	2.00
ASM - R5	2.50	2.00
ASM - R6	3.00	2.33
SWT - R1	1.67	3.50
SWT - R2	2.17	2.00
SWT - R3	1.67	2.17
SWT - R4	1.83	2.00
SWT - R5	1.67	2.00
SWT - R6	1.67	2.00

Table S5: Disease severity index (DSI) of *Botrytis cinerea* (Bc) infection in grape berries treated with *Pseudomonas protegens* strains and their formulations (PP1, PP2, MG, TNR), acibenzolar-S-methyl (ASM), and a cyprodinil + fludioxonil fungicide mixture (SWT). Treatments were applied in a Chardonnay vineyard seven days prior to harvest, followed by artificial inoculation with Bc under field conditions. Disease severity was evaluated after 5 days of incubation in humidity chambers maintained at 22 °C. Six berries were placed in each humidity chamber per treatment, and the experiment was replicated across 16 independent biological replicates per treatment (n = 96). The dataset corresponds to the mean values of observations per treatments. Different letters indicate significant differences according to Tukey's HSD test ($p \leq 0.05$).

Treatment	Disease severity index of Bc mean	Standard error (SE)	Tukey's HSD test ($p \leq 0.05$)
UTC	4.78	0.54	a
PP1	2.78	0.13	b
PP2	3.11	0.30	b
MG	3.17	0.34	b
TNR	2.89	0.21	b
ASM	3.17	0.29	b
SWT	1.78	0.08	c

Table S6: Disease severity index (DSI) of *Botrytis cinerea* (Bc) infection in grape berries treated with *Pseudomonas protegens* strains and their formulations (PP1, PP2, MG, TNR), acibenzolar-S-methyl (ASM), and a cyprodinil + fludioxonil fungicide mixture (SWT). Treatments were applied post-harvest and subsequently inoculated with Bc under laboratory conditions. Disease severity was evaluated after 5 days of incubation in

humidity chambers maintained at 22 °C. Six berries were placed in each humidity chamber per treatment, and the experiment was replicated across 16 independent biological replicates per treatment (n = 96). The dataset corresponds to the mean values of observations per treatments. Different letters indicate significant differences according to Tukey's HSD test ($p \leq 0.05$).

Treatment	Disease severity index of Bc mean	SE	Tukey's HSD test ($p \leq 0.05$)
UTC	4.64	1.03	a
PP1	2.78	0.40	bc
PP2	3.28	0.38	ab
MG	2.14	0.09	c
TNR	2.69	0.39	bc
ASM	2.39	0.24	c
SWT	2.28	0.25	c

Table S7: Powdery mildew disease severity index (DSI) on grapevine leaves evaluated at EL 31 stage (BBCH 75) after four spray treatments. Disease severity was visually assessed using the scale described by Barba et al. [50]: 0 = absent, 1 = <5 small spots, 2 = 5-20 expanding spots, 3 = widespread powdery mildew with dense sporulation. Treatments included untreated control (UTC), MaxGrowth (MG), TANIRI® WP (TNR), acibenzolar-S-methyl (ASM), and sulfur 72% (SUL). Data represent mean DSI values per treatment across six replicates. Fifty leaves per grapevine were randomly selected from three central grapevines within each plot (n = 900). The dataset corresponds to the mean values of observations per replicate.

Treatment	Powdery mildew DSI
UTC - R1	6.11
UTC - R2	4.67
UTC - R3	9.56
UTC - R4	9.56
UTC - R5	8.22
UTC - R6	10.56
MG - R1	3.56
MG - R2	2.89
MG - R3	5.67
MG - R4	5.11
MG - R5	9.67
MG - R6	6.11
TNR - R1	3.44
TNR - R2	3.56
TNR - R3	8.11
TNR - R4	7.22
TNR - R5	6.89
TNR - R6	5.44
ASM - R1	1.56
ASM - R2	1.67
ASM - R3	3.33
ASM - R4	3.00

ASM - R5	4.89
ASM - R6	3.78
SUL- R1	0.56
SUL- R2	0.67
SUL- R3	1.56
SUL- R4	2.00
SUL- R5	2.00
SUL- R6	1.33

Table S8: Powdery mildew disease severity index (DSI) on grapevine leaves evaluated at EL 31 stage (BBCH 75) after four spray treatments. Data represent mean DSI values per treatment across six replicates. Fifty leaves per grapevine were randomly selected from three central grapevines within each plot (n = 900). Mean comparisons were performed using ANOVA, and significant differences among treatments were identified by Tukey's HSD test ($p \leq 0.05$). Different letters indicate significant differences. The dataset corresponds to the mean values of observations per treatments.

Treatment	Powdery mildew DSI mean	SE	Tukey's HSD test ($p \leq 0.05$)
UTC	8.11	0.93	a
MG	5.50	0.97	b
TNR	5.78	0.80	b
ASM	3.04	0.52	c
SUL	1.35	0.26	d

Table S9: Efficacy of control of natural infection of powdery mildew (PM) and Botrytis bunch rot (BBR) on Chardonnay grape bunches by biological and chemical bioinducers after 10 spraying under field conditions. Disease severity on grape bunches, expressed as the percentage of organs with visible signs or symptoms was assessed by visual inspection. Treatments included an untreated control (UTC), MaxGrowth (MG), TANIRI® WP (TNR), and BION® 50 WG (ASM) and Sulfur 72% (SUL). Powdery mildew severity was assessed at the EL 31 stage after four treatments application (n = 180), while *Botrytis cinerea* severity was assessed on harvest grape bunches (EL38) after 7 days of incubation in humidity chambers at 25°C (n = 108). The dataset corresponds to the mean values of observations per replicate.

Treatment	*PM* Disease severity	*BBR* Disease severity
UTC - R1	41.35	67.14
UTC - R2	23.96	83.69
UTC - R3	86.39	83.10
UTC - R4	78.84	92.14
UTC - R5	93.51	77.50
UTC - R6	80.39	85.83
MG - R1	32.16	43.57
MG - R2	43.89	64.40
MG - R3	80.47	88.33

MG - R4	61.27	76.79
MG - R5	85.38	62.50
MG - R6	65.76	63.93
TNR - R1	55.26	43.81
TNR - R2	32.17	73.10
TNR - R3	90.52	87.50
TNR - R4	76.75	57.50
TNR - R5	73.08	59.40
TNR - R6	39.82	42.86
ASM - R1	31.48	40.83
ASM - R2	38.00	59.29
ASM - R3	69.35	67.26
ASM - R4	60.19	53.93
ASM - R5	84.44	76.79
ASM - R6	39.65	44.17
SUL- R1	38.32	47.62
SUL- R2	20.85	51.67
SUL- R3	38.93	92.50
SUL- R4	74.66	60.48
SUL- R5	36.43	31.07
SUL- R6	29.08	25.83

Table S10: Efficacy of control of natural infection of powdery mildew (PM) on Chardonnay grape bunches by biological and chemical bioinducers after 10 spraying under field conditions. Treatments were compared by ANOVA and different letters above the bars denote significant differences among treatments according to Tukey's HSD test ($p \leq 0.05$). The dataset corresponds to the mean values of observations per treatments.

Treatment	*BBR* Disease severity mean	SE	Tukey's HSD test ($p \leq 0.05$)
UTC	67.41	11.41	a
MG	61.49	8.40	ab
TNR	61.27	9.27	ab
ASM	53.85	8.50	bc
SUL	39.71	7.54	c

Table S11: Efficacy of control of natural infection of Botrytis bunch rot (BBR) on Chardonnay grape bunches by biological and chemical bioinducers after 10 spraying under field conditions. Treatments were compared by ANOVA and different letters above the bars denote significant differences among treatments according to Tukey's HSD test ($p \leq 0.05$). The dataset corresponds to the mean values of observations per treatments.

Treatment	*BBR* Disease severity mean	SE	Tukey's HSD test ($p \leq 0.05$)
UTC	81.57	3.47	a
MG	66.59	6.15	ab

TNR	60.69	7.04	bc
ASM	57.04	5.59	bc
SUL	51.53	9.75	c
